# Why Are Hungry College Students Not Seeking Help? Predictors of and Barriers to Using an On-Campus Food Pantry

**DOI:** 10.3390/nu10091163

**Published:** 2018-08-25

**Authors:** Aseel El Zein, Anne E. Mathews, Lisa House, Karla P. Shelnutt

**Affiliations:** 1 Department of Food Science and Human Nutrition Department, University of Florida, Gainesville, FL 32611, USA; aseel.elzein@ufl.edu (A.E.Z.); anne.mathews@ufl.edu (A.E.M.); 2 Department of Food and Resource Economics Department, University of Florida, Gainesville, FL 32611, USA; lahouse@ufl.edu; 3 Department of Family, Youth, and Community Sciences, University of Florida, Gainesville, FL 32611, USA

**Keywords:** food insecurity, food pantry, college, students, barriers

## Abstract

Background: The number of food pantries on U.S. college campuses has increased in response to students’ food insecurity, but limited information is available to describe the impact of this resource. The objective of this cross-sectional investigation was to examine the relationship between food insecurity and food pantry awareness, use, and perceived barriers to use. Methods: Students attending the University of Florida in fall 2017 (*n* = 899) completed the United States Department of Agriculture (USDA) Adult Food Security Survey and responded to questions about food pantry awareness and use. Sociodemographic data were also collected and included in multivariate logistic regression models. Results: While most students (70%) were aware of the existing food pantry, nearly a third of respondents were classified as being food insecure. After adjusting for sociodemographic correlates, factors such as identifying as being food insecure, international status, and receiving a student loan or a need-based federal financial aid (Pell grant) increased the likelihood of utilizing the campus food pantry. Despite these predictors, only 38% of food insecure students reported food pantry use. Among students who provided qualitative insights, four main barriers to using the on-campus food pantry were identified: social stigma, insufficient information on pantry use policies, self-identity, and inconvenient hours. Conclusions: Food security interventions and administrative policy should consider a new model of the traditional campus food pantry that reduces concerns of social stigma and is supported by clear and ongoing communications of operational procedures tailored for the college student population.

## 1. Introduction

A college education is no longer viewed as only an opportunity for financially or intellectually privileged individuals, but also for those hoping to access better financial opportunities [1]. Despite rising college costs [2] and the dwindling purchasing power of forms of need-based financial aid such as the Pell grant program [3], there has been a substantial increase in student enrollment [4], including historically under-represented populations [5]. As a result of these factors, many students experience economic hardships and financial stress [6] that can translate into a budget with significant essential bills (tuition, textbooks, housing), leaving a strain with respect to more flexible expenses, like those allocated to consistent access to adequate and nutritious food [7]. As such, food insecurity, or limited consistent access to nutritionally adequate and safe food, has become a serious public health problem affecting college students in the United States.

Numerous cross-sectional studies have examined the prevalence of food insecurity among college students in the U.S. and reported rates range from 14% to 59% [8,9,10]. A 2017 systematic review of food insecurity among postsecondary students reported an average rate of 42% of college students experiencing food insecurity [11], which is three times higher than the rate for general U.S. households [12]. Food insecurity appears to increase at the end of every semester during the college years [13], and this increase is associated with decreased likelihood of engaging in healthy physical activity habits and increased likelihood of experiencing stress and a depressed mood [13]. Additionally, among college students, food insecurity is associated with eating fewer fruits and vegetables [14], less frequent breakfast intake [15], higher rates of anxiety and depression [16], poorer academic performance [17], and increased difficulty concentrating in classes [18]. Although not examined among college students, food insecure adults report higher rates of diet-related chronic conditions, such as hyperlipidemia, hypertension, and diabetes [19,20], and lower work productivity, compared to their counterparts [21]. Thus, food insecurity may have enduring social and health impacts.

Colleges and universities are addressing students’ food insecurity through the establishment of on-campus food pantries [22]. Food pantries are a type of food assistance service that collects food donations from the community and redistributes food to those in need at no cost. As of May 2018, 640 food pantries were registered in the College and University Food Bank Alliance (CUFBA), a national association that tracks and supports initiatives to reduce hunger, food insecurity, and poverty rates among college students [23]. While on-campus food pantries are considered an easily accessible resource for college students, a 2016 report of 3765 students from 34 college campuses and 12 states indicated that only 14% of food insecure students and their households had utilized a food pantry or food bank in the past month [22].

Widespread establishment of on-campus food pantries is a relatively recent phenomenon [23]. For this reason, peer-reviewed reports describing students’ awareness of existing food pantries, use of food pantries for food acquisition, and their intersection with the students’ food security status, are lacking. It is not known, for example, whether food insecure students are more likely, equally likely, or less likely to use a food pantry or experience barriers to use. Additionally, there is a lack of literature detailing differences in sociodemographic characteristics of students who use a pantry and those who do not, or reports that provide insights on predictors of or perceived barriers to food pantry use by college students. Thus, a clearer understanding of campus food pantry usage and potential barriers to usage in the context of college students’ food insecurity is needed. Findings can inform initiatives and outreach activities to improve the accessibility of on-campus food pantries.

The aims of this study were to (1) examine the awareness and use of an on-campus food pantry by students of varying food security status levels; (2) assess the determinants of the food pantry use; and (3) describe perceived barriers preventing students from utilizing the on-campus food pantry. Findings from this study will help inform potential solutions to combating food insecurity on college campuses, as well as improving on-campus food pantry outreach and use.

## 2. Materials and Methods

### 2.1. Design and Participants

The authors distributed a cross-sectional, non-probability, Web-based survey via email to students attending the University of Florida during October 2017. A total of 899 students completed the survey which was open for a 3-week period. Students across all disciplines and academic years were eligible to participate. The email included online informed consent, and those who accepted consent were directed to the survey. The study protocol was approved by the Institutional Review Board of the University of Florida.

### 2.2. Measures

#### 2.2.1. Food Insecurity

The prevalence and severity of food insecurity were assessed using the ten-item validated USDA Adult Food Security Survey Module (USDA-AFSSM) [24]. The questions cover a range of experiences from worrying that food would run out to not eating for a whole day because of lack of resources to obtain food. In accordance with the USDA Economic Research Service (ERS), participants’ food security levels were determined based on the sum of affirmative responses. As such, students were categorized into one of four food security categories: *high food security* (no food access problems), *marginal food security* (anxiety over food situation), *low food security* (reduced diet quality and variety), and *very low food security* (multiple indications of disrupted eating patterns and reduced food intake) [25].

#### 2.2.2. Food Pantry Awareness, Usage, and Barriers

Students’ awareness of the on-campus food pantry was assessed by asking “Are you aware that the University of Florida (UF) has established an on-campus food pantry to help those who have difficulty in purchasing enough food?” with ‘yes’ and ‘no’ as response options. To identify students as pantry users or non-users, students were asked “Have you visited the campus food pantry for obtaining food?” with ‘yes’ and ‘no’ response options. For students who reported not visiting the food pantry, they were asked whether barriers to use existed. The response options for this question were ‘no, I do not need the food pantry’, ‘no, I do not think there are barriers’, or ‘yes’. Students who indicated the existence of barriers were asked to describe them and responses were analyzed to generate reasons for not using the food pantry. Students who indicated using the food pantry were asked a follow-up question about how they use food from the pantry. Options were that food from the pantry is used ‘to supplement regular food needs’ or ‘as the sole source of food’.

#### 2.2.3. Socio-Demographic Variables

Characteristics such as participants’ sex, race/ethnicity, marital status, year in college, employment, receipt of student loan or Pell grant, place of residence (on-campus/off-campus), and university residence status were collected. Place of residence was assessed by asking students “Where do you currently live?” with ‘on-campus’ and ‘off-campus’ as response options. Students were also asked to identify their university class as freshmen, junior, sophomore, senior, or graduate student. Due to low numbers in some categories, student classification by year was coded as undergraduate or graduate student. Residence status was assessed by asking the students “What is your residence status at the University of Florida?” with three response options that included ‘in-state’, ‘out-of-state’ and ‘international student’. For analysis, race was collapsed into ‘white’ and ‘non-white,’ and ethnicity was reported as ‘Hispanic/Latino’ or ‘non-Hispanic Latino’. Participants were asked to report their marital status using five response options, which were then grouped into ‘single’ or ‘married/partnership’. Age was only assessed by asking the students “Are you 18 years of age or older?”. Data for students who were less than 18 years old were excluded from the analysis. Finally, participants were asked about their preferred food payment method (meal plans, earned income, monthly allowance, scholarships, etc.) and whether they utilized a credit card for food purchases.

### 2.3. Statistical Analysis

Descriptive statistics were computed to describe demographic characteristics. Food security status was determined from the 10 AFSSM questions in accordance with the Guide to Measuring Household Food Security [26]. Based on the protocol, zero affirmative answers indicated high food security, 1–2 indicated marginal food security, 3–5 low food security, and 6–10 very low food security. Prevalence of food insecurity was determined by further collapsing those who scored in the low and very low food secure categories as food insecure and those who scored in the high or marginal food secure categories as food secure. Chi-squared tests of independence were used to determine associations between food security status demographic variables, as well as, food security and food pantry variables, including the awareness of an on-campus food pantry, the existence of barriers limiting its use, purpose of campus food pantry use, and past pantry visits for obtaining food.

We constructed multivariate logistic regression models to evaluate the association between food pantry use and characteristics anticipated to influence the decision or the ability to use the food pantry, including food security status, race/ethnicity, receipt of student loan or Pell grant, marital status, employment, student class (undergraduate/graduate), and university residence status. The model included all significant variables identified using chi-squared analyses or univariate logistic regressions. Results of the logistic regression models were expressed as crude (unadjusted) and adjusted odds ratio (OR) with a 95% confidence interval (CI). The outcome variable was food pantry use, dichotomized to indicate whether a student had ever visited the food pantry for food. All data analyses were conducted using the IBM SPSS Statistics for Windows, version 24 (Armonk, NY); *p*-values < 0.05 were considered statistically significant.

## 3. Results

### 3.1. Study Population Characteristics

The survey was completed by 899 undergraduate and graduate students during the fall 2017 semester. Respondents were predominantly female (74.3%), white (77.6%), non-Hispanic/Latino (82.1%), single (85.6%), and undergraduates (65.6%). Approximately one-quarter of the students (24.3%) reported residing on-campus (Table 1). Almost half of the students (49.7%) reported being employed in either a part-time or full-time job. Thirty-five percent of the students had a student loan, and almost 23% were Pell grant recipients. Mean student self-reported body mass index (BMI) was 23.6 ± 4.8 kg/m^2^. Most (64%) students fell in the healthy (18.5–24.9) BMI category followed in prevalence by the overweight (25.5–29.9) category (21.1%).

### 3.2. Food Security Status

Analysis of the AFSS score showed that 616 respondents (68.5%) were food secure, with 435 of these respondents (48.4%) having high food security and 181 of these respondents (20.1%) with marginal food security. The remaining 283 respondents (32%) were classified as food insecure, consisting of 138 respondents (15.4%) with low food security and 145 respondents (16.1%) with very low food security.

Investigation into the relationship between sociodemographic variables with food security showed significant associations between food security and university residence status (χ^2^ (3) = 17.94, *p* = 0.006), Pell grant (χ^2^ (2) = 43.60, *p* < 0.0001), race (χ^2^ (3) = 33.20, *p* < 0.0001), and student class (χ^2^ (2) = 5.72, *p* = 0.01). Specifically, international students had the highest prevalence of food insecurity (37.6%) when compared to in-state and out-of-state students (30.7% and 29.3%, respectively); undergraduate students were more likely to report food insecurity compared to graduate students (34% versus 26%, respectively); more black (61.7%) students were food insecure than white (27.5%) and other/multiracial (37.8%) students; and Pell grant recipients had double the rate of food insecurity compared to non-recipients (50.2% versus 25.9%, respectively).

### 3.3. Food Pantry Awareness and Determinants of Use

Although most students (70%; *n* = 635) were aware of the existing food pantry (Table 2), only 15.6% had used the food pantry for food acquisition. A significant association was noted between using the food pantry and food security status *(*χ^2^ = 133.2, *p* < 0.0001). Specifically, a higher proportion of food insecure students (low + very low food security) used the food pantry compared to food secure students (high + marginal food security). While food insecure students were more likely to use the pantry than their counterparts, only 38.5% reported using the pantry for food acquisition (Table 2). Students who used the pantry were then asked whether they used the pantry to supplement food intake or as a sole source of food acquisition. Results indicated that more than one-third of students (36.4%) are using the pantry as a sole source of food.

Table 3 presents student characteristics associated with pantry use. This analysis was limited to students who reported being aware of the food pantry (*n* = 635). Results of univariate logistic regression models indicated that food insecure students, non-whites, Hispanic/Latinos, international students, graduate students, students using a credit card to purchase food, and Pell grant or student loan recipients were more likely to use the pantry than their counterparts. These results should be considered carefully though, as some characteristics related to food pantry use are also associated with food security status. To address this issue, multivariate logistic regression models were used, where the dependent variable was the probability of using the campus food pantry. Independent of food insecurity and other sociodemographic characteristics found to be significant in the bivariate analyses, students who received Pell grants (OR: 1.87, 95% CI: 1.01–3.48), received student loans (OR: 2.31, 95% CI: 1.39–3.82), or identified residence status as international (OR: 7.16, 95% CI: 3.13–16.35) were significantly more likely to use the food pantry compared to their counterparts. Results from multivariate analysis also indicated that food insecure students were eight times more likely to use the food pantry compared to food secure students (OR: 8.85, 95% CI: 5.13–15.26).

### 3.4. Barriers to Using the Food Pantry

Among students who did not visit the pantry (*n* = 534), reporting the existence of barriers to use the food pantry was significantly associated with food security status (χ^2^ = 52.61, *p* < 0.0001) (Table 2). A higher proportion of students with low and very low food security (34% and 28.8%, respectively) reported more barriers to use the pantry, compared to highly and marginally food secure students (4.7% and 16%, respectively). A subsample of students who did not visit the pantry provided qualitative answers that yielded four thematic categories based on 68 responses. The main impediments to using the food pantry were social stigma and embarrassment (36.8%), insufficient information on how the program works and what determines eligibility (33.8%), self-identity, or the feeling that the food pantry was not for them (17.6%), and inconvenient hours of operation (11.8%) (Table 4). Half of those reporting social stigma, insufficient information, and inconvenient hours as barriers reported being food insecure.

## 4. Discussion

The utility of programs and interventions to address food security among college students is mostly unknown. Findings from the present study yield important insights into the use of a campus-based food pantry and barriers to its use by college students, considering student food security status. Moreover, our study contributes to the growing literature of student food insecurity in the U.S. by describing the socio-demographic predictors of food pantry use.

We identified that one-third of the responding student population is food insecure, and food insecurity predicted pantry use when controlling for sociodemographic characteristics. However, whether campus-based food pantries are influencing the students’ food security status has not been studied. Longitudinal studies that measure changes in food security status, self-sufficiency, and diet quality of food pantry users are needed. Findings from the present study also shed the needed light on the limited use of the food pantry by food insecure students. Even though food insecure students were more likely to use the pantry than food secure students, most food insecure students were not using the food pantry. Reasons for not using the food pantry were not limited to logistical ones, but also included resistance barriers, including social stigma, and self-identity. Future research should include more qualitative data collection to explore students’ perceived barriers to access and attitudes of food pantry users when seeking food aid from the pantry, including the way they are treated and their perception of the quality and quantity of food provided.

Identifying as an international student predicted food pantry use, and the prevalence of food insecurity among this population was higher than that of both in-state and out-of-state students. The international student population has received little attention when discussing food security despite being exposed to factors that increase their vulnerability to financial hardships. Indeed, in one study of 220 international college students, 56% reported financial difficulties as a source of stress [27]. These challenges are not surprising given that the tuition costs for international students are significantly higher than that of in-state and out-of-state students [28]. For example, while the cost for an in-state student was $212 USD per credit hour at the University of Florida, the rate for an international student was $955 USD per credit hour in 2017 [28]. Additionally, international students are not eligible for domestic loans without a U.S. citizen or a legal permanent resident willing to co-sign for a loan unless they are citizens themselves [29]. Similarly, participation in the Supplemental Nutrition Assistance Program (SNAP) is limited to U.S. citizens although it is difficult to even college students who are U.S. citizens to qualify for SNAP benefits [30]. Finally, the exchange rate between U.S. dollars and domestic currencies, especially for students coming from developing countries, can be high. Collectively, this means that international students likely invest proportionally more of their budget towards the cost of earning a degree in the U.S. These factors may have played a role in the observed high prevalence of food insecurity among international students and encourage further investigation into culturally sensitive strategies among this population.

Reporting the existence of barriers to pantry use was significantly associated with food security status, with a higher proportion of food insecure students reporting barriers to using the food pantry compared to food secure students. Similar to a 2004 investigation of college students’ barriers to seeking food assistance through the food stamp program [31], stigma was the main barrier to using the campus food pantry by University of Florida respondents. This finding suggests the need for an alternative program model to provide food assistance in a less stigmatizing way for students who may benefit most from the pantry and other available resources. One strategy to address the perceptions of pantry use is to employ marketing strategies to “rebrand” the food pantry with input from students in need. For instance, rather than communicating about the food pantry as a resource for those who are in a food-shortage crisis, a food pantry can be a community resource connected with other wellness resources. These resources may include healthy cooking classes on a budget, a point of screening and administering SNAP benefits, and programs designed to improve the students’ food resource management skills, similar to the “Basic Shelf Experience” (used in the U.K.) [32]. This program not only helps educate on the effective use of food resources but also helps individuals cope with food insecurity-related stress. Another example is a novel food pantry intervention in a Connecticut community, called the Freshplace [33]. The latter was a client-choice food pantry intervention that tested the hypothesis that a branded food bank designed to carry less perceived social stigma would result in higher satisfaction and impact on food security status than a traditional food bank. Clients met monthly with a project manager to receive motivational interviewing and track personal goals for becoming food secure in addition to targeted referrals to community services. Results from this one-year intervention indicated that Freshplace intervention had greater acceptability than the traditional food pantry, and its members were less likely to experience very low food security compared with those utilizing the traditional food bank. This intervention could serve as a model for rebranding of food pantries and alleviating stigma while imparting a sense of dignity in seeking food assistance.

Some of the barriers to making use of the pantry identified by our study participants could be addressed by altering food pantry operations. Students identified lack of logistical information on pantry policies and inconvenient pantry hours as reasons for not using the pantry. While on the studied campus all new freshmen receive information about the food pantry during their orientation, details about food pantry operation may not reach students throughout the year when it is needed or through channels that deliver to students struggling with food security. Thus, administrators and staff could increase students’ awareness and knowledge of the pantry operational procedures through communication campaigns tailored to the communication style of today’s students, as well as by extending the food pantry hours. An example of spreading on-campus awareness would be educating student leaders about food insecurity and available food assistance programs. Student leaders can then disseminate information to their student organization members through social media outlets and organization events, broadening awareness and outreach across the college campus. Although these changes require resources, findings suggest that an expansion of operations is necessary for the pantry to have a recognizable impact on college students’ food insecurity.

Even if the campus pantry operations are improved, these improvements may not address the resistance that some participants experienced in seeking help. Students were explicit in their hesitance in receiving charity and expressed that the pantry was meant to serve those with higher needs than their own, thus asserting their identity as self-sufficient, moral individuals [34]. This barrier was previously described among SNAP recipients who avoided the pantry because others needed the food more [35] and among low-income families in Canada [36]. In our study, among students who felt a moral imperative to abstain from using the pantry, one-third were food insecure students who could have benefited from a resource they perceived was needed more by others. Previous research among low-income individuals indicated that such a perception might stem from pantry marketing messages or news stories emphasizing the increased demand for food supplies in food pantries [37]. Thus, outreach efforts in the college settings may require a shift in the marketing of the food pantry.

Nevertheless, traditional food pantries are not intended to solve food insecurity but to provide emergency relief when students run low on financial resources [38]. Despite the lack of literature regarding the effectiveness of on-campus food pantries, research assessing food insecurity among 153 women using food banks in Canada reported that 70% experienced deprivation despite using food banks [39]. The authors concluded that while “food assistance may have alleviated some of the absolute food deprivation in the households studied, it clearly did not prevent members from going hungry” [39]. Therefore, solutions to students’ food insecurity should not be limited to increasing accessibility to campus food pantries. Additional strategies and policies should be considered that may have a broad and sustained impact on food security and associated health and academic issues throughout the college years. Strategies may include indexing Pell grants to the tuition inflation, revising SNAP eligibility for college students [40], extending this amendment to international non-U.S. citizen students who are otherwise not SNAP-eligible, and providing affordable meal plan rates to “at-risk” students who display poverty indicators, including Pell grant recipients. These efforts will require the collaboration of university administrators, student affairs staff, and student organization leaders to advocate for on-campus solutions and decrease the stigma surrounding the use of food assistance resources.

Although our study provided a rare glimpse at the intersection of student food security and food pantry use, this research is not without limitations. First, the cross-sectional nature of this study prevents one from drawing conclusions about causal relationships between food insecurity, barriers to food acquisition, and food pantry use. Second, findings cannot be generalized to college students across the nation as the sample is limited only to a convenience sample of students from one institution. Third, the food insecurity instrument is reliant on student self-reported data, which may have introduced social desirability bias. Additionally, the survey included several skip patterns, that limited the logistic regression analyses to only those who reported being aware of the food pantry (*n*= 635). Another limitation relating to the survey questions is the definition of pantry users versus non-users. Because we defined pantry users as those “having ever visited the food pantry for obtaining food”, without a specific time frame, we were not able to focus on current or frequent food pantry users. Finally, perceived barriers were based on qualitative survey answers of a sub-sample of non-pantry users, which limits probing for potential challenges, barriers, or attitudes that food pantry users experience. Nevertheless, given that no study has examined perspectives on barriers to using a campus food pantry or described predictors of a pantry use, these findings are a valuable contribution to the study of food insecurity and campus food assistance programs.

In conclusion, a significant portion of college students may struggle with hidden hunger, food insecurity, and hesitation in seeking assistance. Findings from this study reveal some insights into perceived barriers to using the campus food pantry. The expressed reasons for not having used the campus food pantry showed difficulty in accessing the pantry and resistance to use despite food insecurity. These issues need to be systematically addressed and findings translated into solutions that focus on reducing stigma, improving outreach and awareness, and increasing pantry access while maintaining students’ confidentiality. Considering the academic [18] and the health-related impact of food insecurity [15], policy-makers and university administrators should prioritize initiatives that successfully address students’ food insecurity. Pursuing a college education should not compete with the right to have consistent access to adequate and nutritious food.

## Figures and Tables

**Table 1:** Characteristics of study population (*n* = 899) ^a^.

Variable	n	%
**Gender**		
Male	230	25.7
Female	664	74.3
**Race**		
White	691	77.6
Black	60	6.7
Asian	116	13.0
American Indian or Alaska Native	2	0.2
Other	23	2.5
**Marital Status**		
Married/Partnership	129	14.4
Single	769	85.6
**Ethnicity**		
Hispanic/Latino	161	17.9
Non-Hispanic/Latino	738	82.1
**Student Year**		
Freshman	158	17.6
Sophomore	119	13.3
Junior	171	19.1
Senior	140	15.6
Graduate	309	34.4
**Place of Residence**		
On-campus	218	24.3
Off-campus	679	75.7
**Pell Grant Recipient**		
Yes	205	22.9
No	692	77.0
**Residence Status**		
In-state	727	81.0
Out-of-state	69	7.7
International	101	11.3
**Employed**		
Yes (full-time/part-time)	446	49.7
No	452	50.3
**Student Loan Recipient**		
Yes	308	34.9
No	575	65.1
**Pay for Food**		
Monthly allowance from parents	370	26.3
Earned Income	479	33.9
Student Loans	175	12.4
Food Stamps	22	1.6
Scholarships	277	19.6
Meal Plans	89	6.3
Other	31	2.1

^a^ Counts will not always sum to 899 because of missing data.

**Table 2:** Prevalence of awareness, use, and barriers to use of the campus food pantry by food security status among college students in the United States, 2017.

Variable	Total *n* (%)	High Food Security (*n* = 435 {48.4%})	Marginal Food Security (*n* = 181 {20.1%})	Low Food Security (*n* = 138 {15.4%})	Very Low Food Security (*n* = 145 {16.1%})	*p*-Value ^a^
**Aware of the food pantry, *n* (%)**						0.05
Yes	635 (70.7)	313 (72.1)	134 (74.0)	99 (71.7)	89 (61.4)	
No	263 (29.3)	121 (27.9)	47 (26.0)	39 (28.3)	56 (38.6)	
**Utilized food pantry b, *n* (%)**						< 0.001
Yes	99 (15.6)	12 (3.8)	15 (11.2)	26 (26.3)	46 (52.3)	
No	535 (84.4)	301 (96.2)	119 (88.8)	73 (73.7)	42 (47.7)	
**Purpose of food pantry visit ^c^, *n* (%)**						
Supplement to regular food needs	63 (63.6)	6 (50.0)	7 (46.7)	18 (69.2)	32 (69.6)	0.27
Sole source of food	36 (36.4)	6 (50.0)	8 (53.3)	8 (30.8)	14 (30.4)	
**Experienced barriers to campus food pantry use**^d^, ***n* (%)**						< 0.001
Yes	68 (12.7)	14 (4.7)	19 (16.0)	21 (28.8)	14 (34.1)	
No	466 (87.3)	287 (95.3)	100 (84.0)	52 (71.2)	27 (65.9)	

^a^ Significantly different at p-value < 0.05; *p*-value was derived using the chi-squared test; ^b^ Question displayed for students who are aware of an existing food pantry; ^c^ Question displayed for students visiting the food pantry; ^d^ Question displayed only for students not visiting the food pantry.

**Table 3:** Predictors of using the campus food pantry by college students in the United States, 2017 ^a^.

Variable	Used Food Pantry (*n* = 99)	Did not Use Food Pantry (*n* = 535)	Crude OR ^b^ (95% CI)	Adjusted OR ^c^ (95% CI)
**Food Security Status, *n* (%)**				
Food Secure	27 (6.0)	420 (94.0)	Ref	Ref
Food Insecure	72 (38.5)	115 (61.5)	9.73 *** (5.97–**15.86)**	8.85 *** (5.13–**15.26)**
**Sex, *n* (%)**				
Male	24 (16.3)	123 (83.7)	Ref	Ref
Female	74 (15.3)	409 (84.7)	1.07 (0.65–1.78)	1.14 (0.64–2.03)
**Ethnicity, *n* (%)**				
Non-Hispanic/Latino	73 (14.0)	449 (86.0)	Ref	Ref
Hispanic/Latino	28 (24.6)	86 (76.8)	1.86 *** (1.12–**3.07)**	1.59 (0.86–2.94)
**Race, *n* (%)**				
White	58 (11.7)	436 (88.3)	Ref	Ref
Non-white	41 (29.3)	99 (70.7)	3.13 *** (1.97–**4.91)**	1.35 (0.75–2.44)
**Housing, *n* (%)**				
On-campus	22 (13.5)	141 (86.5)	Ref	Ref
Off-campus	77 (16.4)	393 (83.6)	1.55 (0.84–2.88)	1.02 (0.57–1.84)
**Marital Status, *n* (%)**				
Not Married	84 (14.9)	479 (85.1)	Ref	Ref
Married/Partnership	15 (21.4)	55 (78.6)	1.53 (0.83–2.84)	1.67 (0.83–3.38)
**International Student, *n* (%)**				
No	75 (12.9)	508 (87.1)	Ref	Ref
Yes	24 (47.1)	27 (52.9)	6.02 *** (3.30–**10.98)**	7.16 *** (3.13–**16.35**)
**Class, *n* (%)**				
Undergraduate	60 (13.5)	386 (61.1)	Ref	Ref
Graduate	39 (21.0)	147 (79.0)	1.70* (1.09–2.65)	1.62 (0.79–3.29)
**Student Loan Recipient, *n* (%)**				
No	47 (11.7)	356 (88.3)	Ref	Ref
Yes	51 (23.0)	171 (77.0)	2.25 *** (1.46–**3.49)**	2.31 *** (1.39–**3.82)**
**Pell grant Recipient, *n* (%)**				
No	62 (12.9)	418 (87.1)	Ref	Ref
Yes	37 (24.3)	115 (75.7)	2.10 *** (1.37–**3.42)**	1.87 * (1.01–**3.48)**
**Employment Status, *n* (%)**				
Not Employed	42 (13.3)	274 (86.7)	Ref	Ref
Employed	56 (17.7)	261 (82.3)	1.40 (0.90–2.16)	1.12 (0.65–1.93)
**Use Credit Card for Food, *n* (%)**				
No	40 (11.9)	295 (88.1)	Ref	Ref
Yes	25 (28.1)	64 (71.4)	2.88 *** (1.63–5.08)	1.46 (0.73–2.92)

Significance is denoted by the following * *p* < 0.05, ** *p* < 0.01, and *** *p* < 0.001; ^a^ Total sample is limited to students who were aware of the existing food pantry; ^b^ Crude OR refers to unadjusted odds ratio of using the on-campus food pantry among the study sample; ^c^ Logistic regression model adjusted for food security status, receipt of Pell grant, student loan status, student class, race/ethnicity, employment, and university residence status.

**Table 4:** Thematic analysis of qualitative data on barriers to using the campus pantry by college students in the United States, 2017.

Theme	Related Quotes
Social stigma	I would feel intimidated to walk in because of the stigma around food insecurity Swiping in at the door is too visible, I am ashamed It is embarrassing for people to know you don’t have enough money for food Fear of judgment I would be embarrassed to be seen there You don’t want others to see that’s where you get your food from Stigma from people seeing you go in and out There may be a social stigma associated with it I believe it’s for students having a difficult time obtaining food, so a lot of students try to stay away because they do not want other students to know
Information	I do not know how the food pantry works Not enough information about eligibility to go there I do not know the hours of operation or what I have to do to get food Unclear hours and procedure {I am} not sure where it is I don’t know if it is available for anyone who walks in {I am} not sure if I qualify Unclear procedure Who’s eligible to go? Where is it located? How does the process work? {I} would like more information on pantry’s expectation of students’ financial situation to use the service
Self-identity	I would be taking resources from those who may need it more than me While sometimes I do not have enough money to buy food, I also feel that I am not poor enough I feel that I want to use the services, but I do not feel that my situation is any worse than other college students I never wanted to take away from people that I know may need more than I do so I never considered going
Hours of operation	Hours interfere with the class schedule Time open is a barrier. I have to go after work hours. Hours are unaccommodating Work and school schedule interfere with opening hours I have two kids and the only time allows me to visit is when they are out of school. I have not thought that it is a realistic situation
